# Proteins with Complex Architecture as Potential Targets for Drug Design: A Case Study of *Mycobacterium tuberculosi*s

**DOI:** 10.1371/journal.pcbi.1002118

**Published:** 2011-07-21

**Authors:** Bálint Mészáros, Judit Tóth, Beáta G. Vértessy, Zsuzsanna Dosztányi, István Simon

**Affiliations:** 1 Institute of Enzymology, Hungarian Academy of Sciences, Budapest, Hungary; 2 Department of Applied Biotechnology, Budapest University of Technology and Economics, Budapest, Hungary; University of Heidelberg, Germany

## Abstract

Lengthy co-evolution of *Homo sapiens* and *Mycobacterium tuberculosis*, the main causative agent of tuberculosis, resulted in a dramatically successful pathogen species that presents considerable challenge for modern medicine. The continuous and ever increasing appearance of multi-drug resistant mycobacteria necessitates the identification of novel drug targets and drugs with new mechanisms of action. However, further insights are needed to establish automated protocols for target selection based on the available complete genome sequences. In the present study, we perform complete proteome level comparisons between *M. tuberculosis*, mycobacteria, other prokaryotes and available eukaryotes based on protein domains, local sequence similarities and protein disorder. We show that the enrichment of certain domains in the genome can indicate an important function specific to *M. tuberculosis*. We identified two families, termed pkn and PE/PPE that stand out in this respect. The common property of these two protein families is a complex domain organization that combines species-specific regions, commonly occurring domains and disordered segments. Besides highlighting promising novel drug target candidates in *M. tuberculosis*, the presented analysis can also be viewed as a general protocol to identify proteins involved in species-specific functions in a given organism. We conclude that target selection protocols should be extended to include proteins with complex domain architectures instead of focusing on sequentially unique and essential proteins only.

## Author Summary

*Mycobacterium tuberculosis* (MTB), the causative agent of TB, is a dramatically successful pathogen that poses a considerable challenge for modern medicine. The increase in multi-drug resistant TB necessitates the identification of novel drug targets and drugs with new mechanisms of action. In this work, we developed a novel computational strategy based on comparative proteomic analysis that can highlight proteins involved in specifies-specific functions. Our analyses of the proteins encoded by the MTB genome identified two protein families that stand out in this respect. These proteins have complex architecture combining various domains and disordered segments. They are also involved in vital functions, especially in host-pathogen interactions. Although these proteins generally do not fit into traditional drug design paradigms, there are several new strategies emerging that can be used to target these proteins during drug development. Our results challenge current target selection protocols that largely rely on the uniqueness and the essentiality of proteins. Instead, these findings emphasize the importance of complex evolutionary scenarios that can lead to the emergence of species-specific functions from more ancient building blocks of proteins. The experiences gained from this work have important implications specifically for targeting MTB, and in broader terms, to improve current target selection protocols in drug development.

## Introduction

Tuberculosis (TB) remains a major world-wide health hazard, causing to roughly 2 million deaths per year. Approximately, one third of the world's population is currently infected with *Mycobacterium tuberculosis* (MTB), the causative agent of TB [1], [2]. MTB is an intracellular parasite, an organism notoriously hard to fight. One of the major reasons for its persistence is the intricate network of host-pathogen interactions which is exploited by the bacterium and which creates a fine-tuned niche for its survival in macrophages [3]. This has been developed during lengthy periods of “co-habitation” and, consequently, co-evolution. The MTB genome has been molded to accommodate the circumstances of life within macrophages. In fact, the bacterium has been so successful in this process that it is notably hard to cultivate outside its physiological host. During the co-evolution process with humans (cf. archeological data presenting experimental evidence for the co-habitation of MTB and humans back to 9000 years [4]), the genome changes within the bacterium have been facilitated by its error-prone DNA polymerases [5]. As a result, the present MTB organism is very close to being an obligatory intracellular parasite.

Mycobacteria are intrinsically resistant to most commonly used antibiotics and chemotherapeutic agents. Due to its specific structure and composition, the mycobacterial cell wall is an effective permeability barrier, generally considered to be a major factor in promoting the natural resistance of mycobacteria. Only a few drugs are active against mycobacterial pathogens, and current treatment strategies for TB consists of 3 or 4 drugs used in combination. However, the increasing emergence of multi-drug resistant tuberculosis (MDR-TB) and extensively drug-resistant tuberculosis (XDR-TB) necessitates the development of novel drugs [6]. Furthermore, novel drugs compatible with antiretroviral therapy are needed to treat co-infected AIDS patients [7] and new drugs are also required that can specifically be employed for children. Clearly, there is an urgent need for drug development projects that actually possess novel targets and novel mechanisms of action [8].

A significant step towards understanding the biology of MTB was provided by full genome sequencing of various strains of this microorganism, including the best characterized laboratory strain, H37Rv, that contains 3,984 genes [9]. The complete genome sequences of several other mycobacteria have also become available, showing various levels of divergence [10], [11]. While the genome size of *M. bovis* is largely similar to that of MTB, the genome of *M. leprae* is reduced to only 40% of that of MTB [12]. These genomes can also be compared to those of many other pathogenic and non-pathogenic bacteria, as the number of fully sequenced bacterial genomes is over 600 and is rapidly increasing. The genomes of several eukaryotic organisms have also been sequenced and are now largely annotated, including the human genome. Additionally, the Human Microbiome Project (HMP) has published the sequenced genomes of 178 microbes that exist within or on the surface of the human body [13], [14]. The plethora of genomic sequences offers a novel platform for comparative analyses and large-scale studies. This new source of data can help to identify proteins in the MTB proteome that perform essential functions ensuring the survival and virulence of the bacterium. These proteins present potential targets for drug design.

Target selection is the crucial starting point of any drug development process. Traditionally, this procedure relied on established knowledge of individual proteins and their functions. The availability of complete genome sequences opened a new era and lead to the development of various bioinformatics methods which can prioritize targets in an automated cost-effective way. These approaches can take various criteria into account with the aim to minimize the interactions with the host environment yet specifically attack the pathogen's growth and survival. Several such studies focused on metabolic enzymes. In their work, Anishetty and co-workers collected enzymes from the biochemical pathways of MTB using the KEGG metabolic pathway database [15]. As a result, 186 proteins were suggested as potential drug targets based on the lack of similarity to proteins from the host *H. sapiens*. Hasan and co-workers proposed a ranking system by targeting metabolic checkpoints based on the uniqueness of their role in the pathogen's metabolome [16]. Additionally, targets were penalized for having high sequence similarity to proteins of the host and of the host flora. The targetTB database was created based on similar principles [17]. Using flux balance analysis and network analysis, proteins critical for the survival of MTB were first identified, and then subjected to comparative genomics analysis with the host. Finally, a novel structural analysis of potential binding sites was carried out to assess the validity of a protein as a target. The selection also incorporated data about the essentiality of proteins using the results of experiments carried out under nutrition rich conditions. A recent analysis constructed a proteome-wide drug target network by linking the structural proteome of MTB with structurally characterized approved drugs [18].

In most drug target selection protocols, the existence of a protein structure or a structural homologue is treated as an advantage for rational drug design. Breaking with this tradition, Anurag and Dash suggested a list of intrinsically disordered proteins in the MTB genome as potential drug targets [19]. This is in accordance with the recent finding that these proteins can also serve as promising drug targets [20], exemplified by the successful blocking of the p53-MDM2 interaction by a small molecule [21]. Fueled by this observation, a list of proteins with disordered protein segments were compiled and filtered for essentiality, uniqueness and involvement in protein-protein interactions. This resulted in 13 proposed drug targets. These proteins have a probable role in signaling, regulation and translation, instead of metabolisms [19].

The success of the target selection procedure critically depends on identifying distinctive features of the pathogen that are essential for its survival. The protein repertoire encoded by the genome provides the initial starting set from which potential targets can be selected based on various hypotheses. However, the optimal target selection criteria are still a matter of considerable debate [22], [23]. The prime criteria of current target selection protocols are essentiality, lack of sequence homologues at least in the host, and the presence of functional characterization. These criteria, however, can lead to the overlook of several important candidates. In the case of MTB, there are several proteins that do not meet the aforementioned criteria but should not be disregarded as potential targets due to their eminent biological importance. For example, the genome sequence of MTB revealed that about 10% of the coding of the genome is devoted to two largely unrelated families of acidic, glycine-rich proteins, the PE and PPE families [9]. These proteins are largely sequence specific to mycobacteria and have been implicated in host-pathogen interactions and antigenic variations [24]. However, most of these proteins are not essential and their function is largely uncharacterized. An additional new class of promising targets in MTB corresponds to signaling elements, in particular to the pkn family of Ser/Thr protein kinases [25], [26]. These MTB proteins play essential roles in both bacterial physiology and virulence [26], but are evolutionary related to eukaryotic protein kinases. These protein families are cases that challenge current target selection protocols and indicate that different approaches for target selection are needed.

In this work, we propose a novel computational strategy based on phylogenetic profiling and comparative proteomic analysis that can highlight proteins involved in specifies-specific functions. This approach takes into account the complex evolutionary scenarios that can lead to the emergence of novel species-specific functions. Novel function can arise from de-novo protein creation but also from more ancient proteins by the combination of divergence, duplication and recombination events [27]. In order to gain insights into the contribution of the various processes, we carried out a comparative proteomic analysis. By focusing on the causative agent of tuberculosis, we analyzed the protein domain and disorder content of its proteome and carried out large-scale local sequence similarity searches to identify basic evolutionary patterns in MTB. We show that the enrichment of certain protein families in the genome can automatically indicate an important function specific to this pathogen. The implications of these findings for target selection are also discussed.

## Results/Discussion

### Comparative sequence analysis of the MTB proteome

#### Domain composition and disorder content of MTB proteins

Domains represent the evolutionary building blocks of proteins. They correspond to conserved regions of proteins with generally independent structural and functional properties. Proteins can be highly modular and contain different domains [28]. The occurrence of different domains can be highly characteristic of the organism [29]. We analyzed the domain composition of MTB in order to identify distinctive features as compared to other organisms.

**Figure 1:**
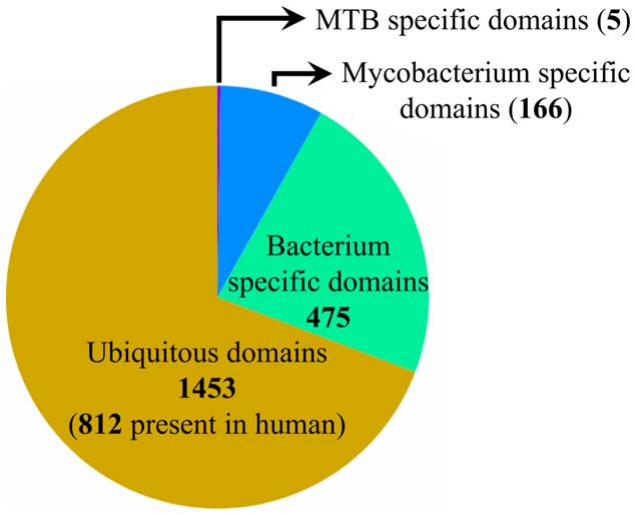
Occurrences of domains of *M. tuberculosis* in other organisms. The distribution of the 2099 Pfam domains present in the proteome of MTB in Eukaryotes and Bacteria. Slices of the pie chart correspond to different levels of specificity with purple showing domains that can be found exclusively in MTB, blue and green showing domains found in mycobacteria or in bacteria in general, respectively and orange showing ubiquitous domains that can be found in organisms from MTB to eukaryotes. Numbers of domains are given for each slice, with number in parenthesis for ubiquitous domains showing the number of domains present in human proteins.

For the definition of domains the Pfam database was used (see Methods) [30]. Scanning the MTB proteome against the Pfam domains revealed that the 3948 MTB proteins altogether contain 5361 instances of 2099 different domains (1592 Pfam-A and 507 Pfam-B domains) with more than 87% of the 3948 MTB proteins containing at least one instance of a domain. Figure 1 shows the occurrence of these domain types in two kingdoms of life (Eukaryote and Bacteria). It can be seen that more than two thirds of the occurring domain types are ubiquitous and can be found in both kingdoms of life and more than half of them can be found in the human proteome as well. The majority of these domains can also be found in archaeal proteins (data not shown). The second largest group of domains totaling about one quarter of all domains cannot be found in eukaryotes but are wide-spread among bacteria in general. These data indicate that a large portion of the genome of MTB is common to many different organisms pointing to their shared evolutionary history. Only about 166 of the occurring domains are specific to mycobacteria and only 5 of the domains were found to be specific for MTB alone. Nevertheless, existing Pfam domains only cover about 63% of all residues (834,389 out of 1,327,431).

Pfam domains are defined based on their evolutionary conservation and generally correspond to globular structures [31]. Proteins can also contain disordered segments that do not adopt a well-defined structure [32]. These regions can serve as domain linkers and therefore contribute to complex domain architectures [33]. Furthermore, they can also participate in binding to other macromolecules via a process that usually involves a disorder-to-order transition [34]. Disordered regions generally have distinct sequence properties and can be predicted from the amino acid sequence [35], [36]. Recently, a method called ANCHOR that can recognize specific regions that are disordered in isolation but can undergo a disorder-to-order transition has been also suggested [37]. The evolutionary analysis of these sequences, however, remains challenging, due to the compositional bias and low complexity of these sequences [38].

We calculated the amount of protein disorder using IUPred [39], [40] and the amount of disordered binding regions using ANCHOR [37], [41] (see Methods). At the residue level, 11.8% and 5.7% of residues were predicted to belong to a disordered segment or a disordered binding region, respectively. Although these values were relatively small, they represented significantly higher values compared to many other bacteria. The fraction of disordered proteins and disordered binding regions were even comparable to that of simpler eukaryotes [19] [Mészáros *et al.* in preparation]. Pfam domains and disordered regions characterize two different aspects of proteins (sequence conservations vs. structural state). Nevertheless, they tend to overlap less than it is expected by chance. Only 7.2% of the positions with corresponding Pfam annotations were predicted as disordered, in contrast to the expected 11% in the random case. This difference is statistically significant. Among the positions belonging to disordered regions, 38.6% belonged to Pfam domains. Therefore, Pfam domains and disordered segments are largely complementary to each other, although some overlap can occur.

Altogether, 28% of the residues of MTB proteins were not covered by either Pfam domains or by disordered and disordered binding regions. Most of these regions are expected to be specific to MTB. However, the coverage of known domains can also be limited by technical difficulties. For example, current methods used for the identification of conserved domains may fail to recognize distant sequence similarities between proteins form different organisms. Additionally, these methods are also limited by the availability of similar sequences. This effect is expected to diminish as the number of complete genomes sequences is increasing, as these novel sequences can help to bridge over missing evolutionary links. Indeed, a large number of Pfam-B domains formed by completely uncharacterized proteins suggest that there are many protein domains waiting to be discovered and characterized.

#### Categorization of MTB proteins based on their specificity and function

We also carried out a large-scale sequence similarity search for all proteins in MTB by comparing them to the proteomes of a wide range of other organisms. By virtue of this analysis, the number of homologs in other bacterial or eukaryotic proteomes was determined for each protein present in MTB. This allows the identification of MTB specific proteins at various levels, as well as the collection of proteins and protein segments that are enriched in MTB.

In order to evaluate these results, proteins were grouped according to their level of evolutionary specificity. At the first level, proteins that were specific to MTB and the highly similar *M. bovis* were compiled. Proteins that occur only at the level of mycobacteria comprised the second level. The third level contained proteins that could be found in other bacteria as well. The last and the largest group included those proteins that were ubiquitous from mycobacteria to eukaryotes. These groups are mutually exclusive, accordingly, each of the 3,948 MTB proteins were classified in one and only one group based on the number of similar sequences in other organism groups. We also analyzed how these proteins were distributed among various functional categories. Functional categorization was obtained from the TubercuList database [42]. Based on this database, proteins were assigned to one of nine functional classes (see Methods).

**Figure 2:**
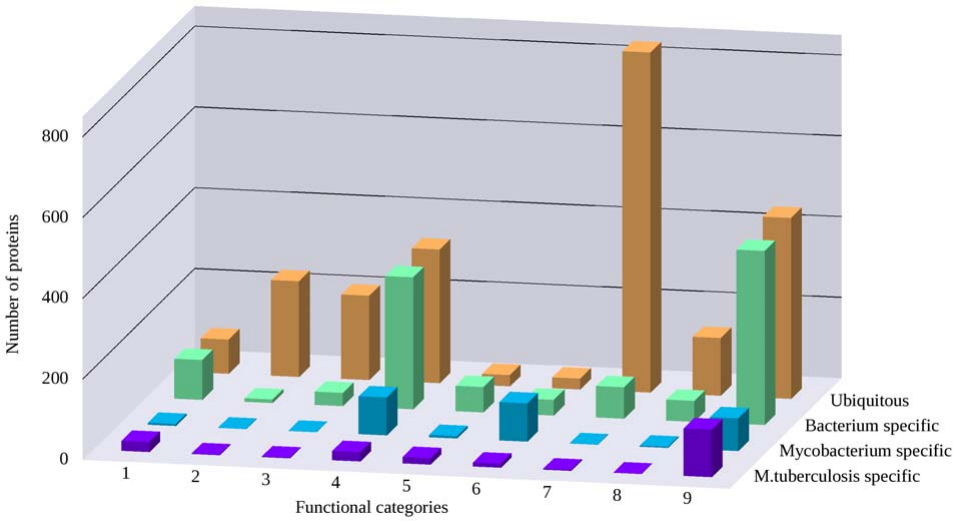
MTB proteins categorized by their functions and their level of specificity. MTB proteins categorized by their functions and their level of specificity. Specificity was defined based on the similarity searches in other, bacterial and eukaryotic proteomes. Proteins that do not show significant similarity to any proteins outside the MTB or *M. bovis* proteomes are considered “MTB specific” (purple), proteins with homologs in other mycobaceria, other bacteria are labeled accordingly (blue and green bars). Ubiquitous proteins with homologues in all kingdoms of life are shown with orange bars. As both functional categories and specificity levels are mutually exclusive, the sum of all bars is equal to the total number of MTB proteins. Functional categories are numbered as follows: 1 – virulence, detoxification, adaptation; 2 – lipid metabolism; 3 – information pathways; 4 – cell wall and cell processes; 5 – insertion sequences and phages; 6 – PE/PPE; 7 – intermediary metabolism and respiration; 8 – regulatory proteins; 9 – conserved hypotheticals.

Figure 2 shows how various proteins are distributed at the different levels of specificity and functional categories. Considering the distribution of proteins among different levels of specificity the results are consistent with the evolutionary incidence of Pfam domains present in MTB (see Figure 1). The majority of the proteins (over 59%) are ubiquitous and even have relatives among eukaryotic proteins. 29% of MTB proteins are unique to bacteria but only 7% and 5% are unique to mycobacteria and to MTB together with *M. bovis*, respectively.

Proteins from the nine studied functional categories defined in the TubercuList (see Methods) exhibited strikingly different distributions among different levels of specificity. One of the largest functional group, corresponding to “intermediary metabolism and respiration”, as well as proteins involved in “lipid metabolism”, “information pathways” or “regulation” essentially lack MTB specific proteins and are overwhelmingly dominated by proteins that have homologs in eukaryotes. This is in agreement with the universality and ancient origin of proteins involved in these processes. Other functions that could be expected to mostly contain proteins unique to MTB such as “cell wall and cell processes”, “insertion sequences and phages” and even “virulence, detoxification and adaptation” include proteins from all levels of specificity. In these cases, however, the contributions from bacterium specific proteins are much larger. This shows that a significant part of these processes are general to all organisms and this shared functional background is modulated in bacteria, mycobacteria and in MTB separately to various extents. However, this modulation is significant in MTB even compared to mycobacteria in general. Correspondingly, these three categories contain a large fraction of MTB specific proteins. A distinct functional class is presented by the “PE/PPE” proteins. This group stands out from other functional groups because most of the proteins in this group are specific to mycobacteria in general. The largest functional category, however, corresponds to the “hypothetical conserved proteins” for which very little information is available. As the majority of MTB specific proteins still fall into this category, this observation cautions that we are only at the beginning to understand the biology of MTB. However, the number of these proteins is expected to decrease as more and more genomes are being sequenced and functionally annotated. For example, 22 out of the total of 1074 conserved hypothetical MTB proteins have a highly similar homolog in the recently characterized *M. pneumoniae* proteome [43]–[45]. Despite these similarities, *M. pneumoniae* does not contain any PE/PPE proteins. Altogether, figure 2 shows that the various functional categories rely on species-specific proteins to a different extent. Interestingly, even those functional groups that are expected to be more specific to MTB are dominated by proteins that have homologues in a wide range of other organisms.

#### MTB specific proteins vs. MTB specific processes

To explore the relationship between MTB specific proteins versus MTB specific processes from a different angle, we selected a mycobacterium specific process, the synthesis and processing of mycolic acids. Takayama et al. analyzed the mycolic acid pathway and described 42 proteins that can be linked to this process [46]. We have collected the domains occurring in these proteins to see how unique its building blocks are to mycobacteria (Table S1 shows these proteins together with the found Pfam domains and the occurrences of these domains in other organisms). The 42 proteins contain 78 occurrences of 37 different Pfam domains. The analysis of the occurrences of these domains in other organisms showed that with the exception of 5 domains, all of them can be found in numerous bacterial and eukaryotic proteins. Most of these domains are also present in various human proteins. Although these proteins carry out an essential function specific to MTB, their sequence cannot be considered specific to mycobacteria or any other group of bacteria.

Four out of the five remaining domains are of unknown function (Pfam-B_2395, Pfam-B_27575, Pfam-B_25573 and Pfam-B_30948). Although these domains do not occur in eukaryotic proteins, other parts of the MTB proteins containing them are much less specific in their sequence. These regions contain commonly occurring domains and show local sequence similarity to bacterial and eukaryotic proteins. Only 1 of the 42 examined proteins can be considered entirely specific to mycobacteria, the largely uncharacterized FAS-II protein (UniProt ID: P64685). This protein solely constitutes of the DUF2662 domain, nevertheless, very little is known about its function apart from the fact that it is involved in fatty acid biosynthesis and has acyl-CoA thioesterase activity. These findings suggest that organism specific processes are not necessarily brought about by organism specific proteins. The discrepancy between the specificity of proteins and the specificity of processes that involve them can be resolved by considering that protein sequence and structure conservation (which is the basis of the definition of domains) does not necessarily result in functional conservation. Proteins with essentially the same fold can carry out different functions as exemplified by many enzymes. Furthermore, the combination of domains with relatively aspecific functions can result in highly specific processes and pathways. For example the MTB ACP protein (Uniprot ID: P0A4W6) contains one acyl-carrier domain (Pfam name: PP-binding) and plays a role in meromycolate extension. The same domain can be found in bacteria and eukaryotes as well, even in human proteins. The human mitochondrial NDUFAB1 protein also contains this domain but plays a different role as the carrier of the growing fatty acid chain during biosynthesis.

#### Similarity based clustering of MTB proteins

**Figure 3:**
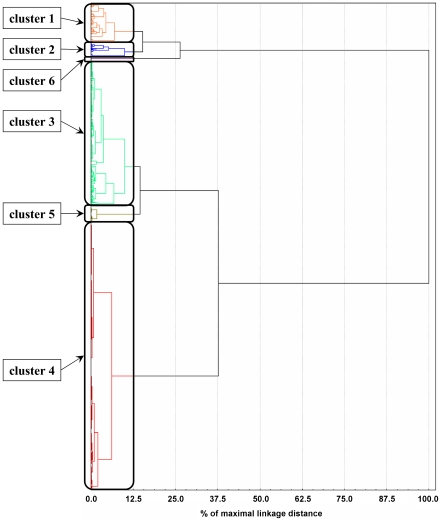
Clusters of MTB proteins based on local protein similarities. Hierarchical tree representing the clustering of the 3,948 MTB proteins using their similarity profiles (see Methods). The tree was cut at 12.5% of the maximal linkage distance and the resulting 6 clusters were analyzed.

**Figure 4:**
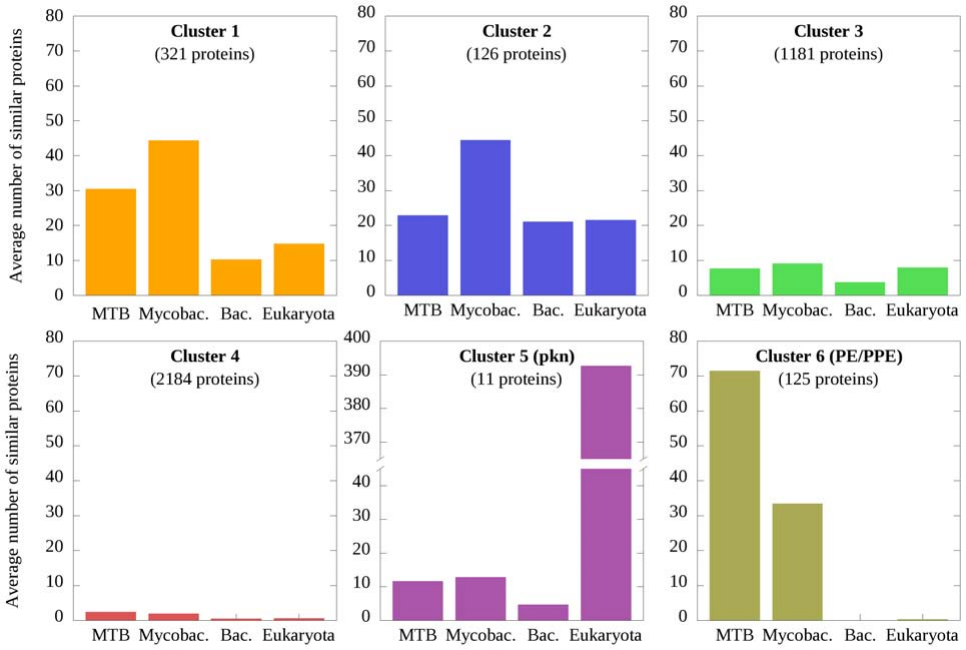
Average similarity numbers for each of the 6 clusters of MTB proteins. Average number of sequences similar to MTB proteins in 4 groups (MTB, mycobacterial, bacterial and eukaryotic proteomes) calculated separately for the 6 clusters resulting from the cluster analysis.

Generally, the number of sequences similar to an MTB protein sequence across various species can show quite large variations. The various scenarios include organism-specific proteins, nearly ubiquitous proteins for which the number of homologs is relatively constant from bacteria to eukaryotes, and many other cases for which significant enrichment/depletion of certain protein families can be seen at certain points in evolution. In order to identify some of the basic trends, we carried out a cluster analysis of the similarity profiles of MTB proteins (see Methods and Table S2). Our analysis focuses on the enrichment and depletion of protein segments in the MTB proteome across different species. In accordance, the similarity profiles were constructed using the number of similar sequences in the proteome of other organisms of each MTB protein. This is in contrast with the binary profiles commonly used in phylogenetic profiling [47]–[49]. The result of the clustering is represented as a hierarchical tree shown in Figure 3. The tree can be dissected into 6 major branches, grouping the MTB proteins into 6 distinct clusters. The complete lists of proteins in each cluster are given in Table S3. For each cluster, we calculated the number of homologs at four levels: within MTB, in mycobacteria, in other bacteria and in eukaryotes on average. These data, shown in Figure 4, provide a clue to decode the basic differences among the clusters.

The distribution of proteins in these clusters is not even, cluster 3 and 4 contains the majority of MTB proteins, while cluster 5 is the smallest with only 11 members. Four of the six clusters, clusters 1–4 show a relatively even distribution in the average number of similar sequences in MTB, mycobacterial, bacterial and eukaryotic proteomes (see Figure 4). The main difference between these clusters is the absolute number of homologs in each group. This is the largest in cluster 1 and smallest in cluster 4. Cluster 4 contains basically all mycobacterium specific proteins. However, many other proteins that have only a small number of similar sequences in all organisms are also part of this cluster. Interestingly, the average number of similar sequences in bacterial proteomes is smaller than in either MTB or mycobacteria for each cluster. This indicates that many proteins, although they share a common evolutionary origin with other bacterial proteins are specifically enriched in MTB. The largest differences in the number of similar sequences in mycobacteria compared other bacteria can be observed in the last two clusters. Cluster 6 represents a group of proteins that are present in MTB in a large number, but completely missing from bacteria other than mycobacteria and are generally not present in eukaryotes either. Members of cluster 5 have around 10 members within MTB and other mycobacteria on average, slightly more than in bacteria in general, but the number of similar sequences of these proteins explodes in eukaryotes with an average count over 390.

The clusters also differ in terms of their functional categorization (Table 1). Each cluster can be reduced basically to three dominant functional classes (marked with bold in Table 1). However, there is a single category that contains at least 30% of the proteins for each group (marked with cyan). The most dominant functional category for cluster 1 is “lipid metabolism”, for cluster 2 is “cell wall and cell processes”, and “intermediary metabolism and respiration” for cluster 3. Cluster 4 – which contains most of MTB specific proteins – is dominated by “conserved hypothetical proteins”. The remaining two groups are homogeneous in terms of their function, cluster 5 contains only regulatory proteins while cluster 6 corresponds to PE/PPE proteins. At a closer look, both of these clusters correspond to a specific family of proteins. The smaller compact cluster of regulatory proteins coincides with the pkn family. Members of the pkn group are distinguished from other MTB proteins in that a large number of similar sequences occur in eukaryotic proteomes, while having only a few or no similar sequences in bacterial proteomes. Members of the other, PE/PPE group contain one of the defining domains (either the PE or PPE domains, or both) which are exclusive to mycobacteria. A few members of this family contained other domains that, in contrast to the defining domains, were not specific to MTB but occurred also in other bacterial or eukaryotic proteins. Depending on this, 25% of PE/PPE proteins belonged to other clusters.

**Table 1:** Functional distribution of proteins in the 6 identified clusters.

Cluster ID	1		2		3		4		5 (pkn)		6 (PE/PPE)	
cell wall and cell processes	23	7.2%	**45**	**35.7%**	*182*	*15.4%*	*521*	*23.9%*	0	0%	0	0%
intermediary metabolism and respiration	*67*	*20.9%*	*40*	*31.7%*	**464**	**39.3%**	*349*	*16.0%*	0	0%	0	0%
lipid metabolism	**128**	**39.9%**	9	7.1%	74	6.3%	34	1.6%	0	0%	0	0%
information pathways	1	0.3%	1	0.8%	78	6.6%	161	7.4%	0	0%	0	0%
regulatory proteins	*61*	*19.0%*	*28*	*22.2%*	56	4.7%	39	1.8%	**11**	**100%**	0	0%
virulence, detoxification, adaptation	12	3.7%	2	1.6%	57	4.8%	139	6.4%	0	0%	0	0%
PE/PPE	0	0.0%	0	0.0%	10	0.8%	32	1.5%	0	0%	**125**	**100%**
insertion seqs and phages	0	0.0%	0	0.0%	57	4.8%	52	2.4%	0	0%	0	0%
conserved hypotheticals	28	8.7%	1	0.8%	*201*	*17.0%*	**844**	**38.6%**	0	0%	0	0%
unknown	1	0.3%	0	0.0%	2	0.2%	13	0.6%	0	0%	0	0%
**Total**	**321**	**100.0%**	**126**	**100.0%**	**1181**	**100.0%**	**2184**	**100.0%**	**11**	**100%**	**125**	**100%**

Distribution of proteins according their functional categories for the 6 identified clusters. Numbers in italics indicate the dominant functions in each cluster and bold typesetting marks the most abundant function.

The disorder content and the amount of disordered binding regions were also distributed highly unevenly among the 6 clusters. Table 2 shows the average fraction of residues predicted to be in disordered or disordered binding regions together with the average length for each of the 6 clusters and for the whole MTB proteome. The two clusters corresponding to two specific proteins families – the pkn and PE/PPE families - had a significantly higher content of protein disorder and disordered binding regions compared to the other clusters and in MTB proteins in general.

**Table 2:** Amount of disorder in the 6 identified clusters.

Cluster ID	Number of proteins	Average protein length	Fraction of disordered AA	Fraction of AA in disordered binding regions
1	321	468	6.04%	3.18%
2	126	371	5.92%	3.28%
3	1181	415	8.68%	5.16%
4	2184	260	13.60%	8.70%
5 (pkn)	11	620	24.17%	17.88%
6 (PE/PPE)	125	514	35.02%	11.69%
**Total MTB**	**3948**	**336**	**11.76%**	**6.77%**

Distribution of residues in disordered and disordered binding regions in the 6 identified clusters.

The two compact clusters corresponding to pkn and PE/PPE families stand out in several respects. The background frequencies of the domain occurrences differ in the two cases, as the homologs of the pkn family is more common in eukaryotes, while members of the PE/PPE family are basically mycobacterium specific. However, both of these groups show a drastic domain enrichment in MTB Beside their very unusual evolutionary profiles, they also exhibit high disorder content. Both of these properties could indicate their functional importance. Further insights can be gained by looking at the functional and structural properties of these two families in more detail.

#### pkn protein family

Members of the pkn family belong to the group of eukaryotic-like Ser/Thr protein kinases (STPKs) [25], [50]. Originally these proteins were thought to be unique to eukaryotes, however, the accumulation of genomic sequences revealed that some prokaryotes also contain members of this group. The bacterial signaling pathways usually rely on two-component systems, basically consisting of a sensor histidine kinase and a response regulator. The eukaryotic-like protein kinase genes, however, represent an independent, additional mode of bacterial regulation. In mycobacteria, genome sequence data indicate that the number of STPK genes is in fact either commeasurable or even considerably higher than those representing the usual bacterial two-component system genes [26]. In the MTB genome, 11 STPK genes can be identified (from pknA to pknL) (Table S4).

**Figure 5:**
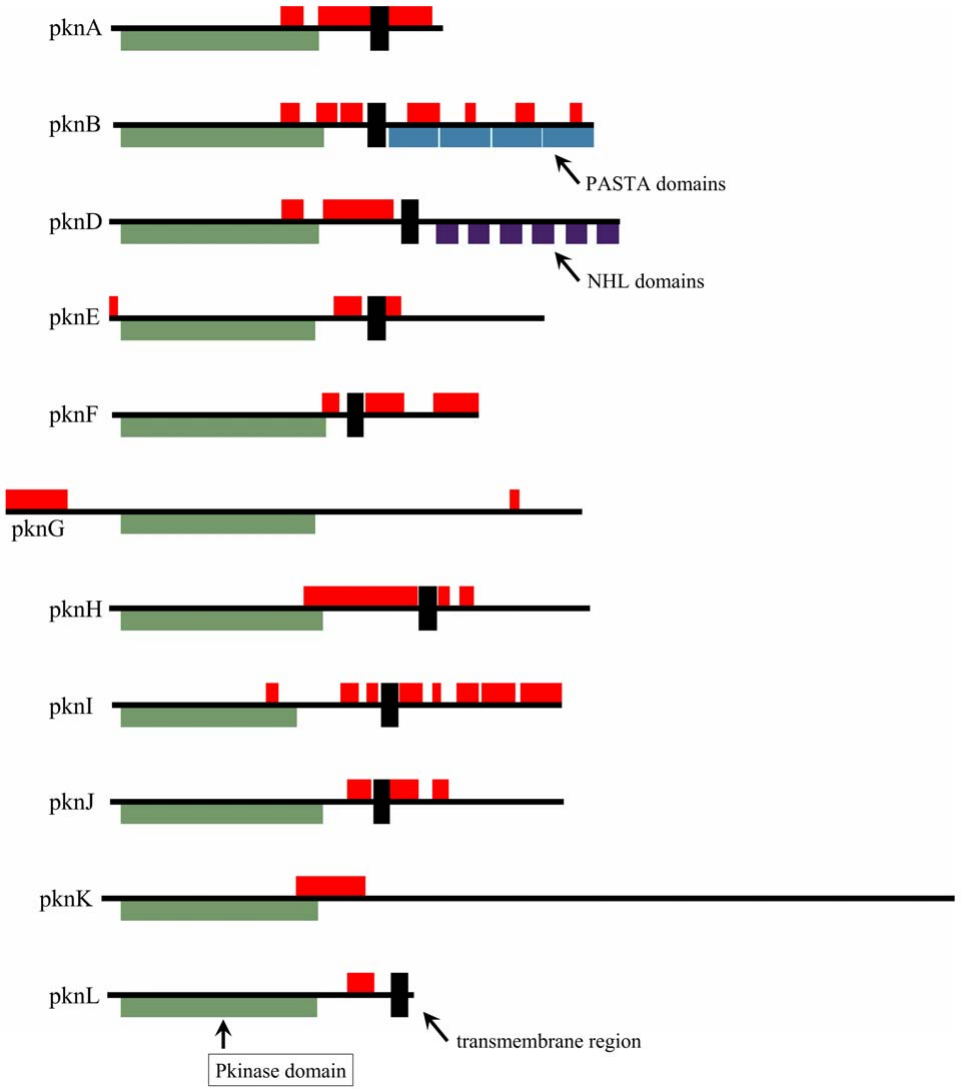
pkn protein domain architectures. Domain architecture of the 11 members of the pkn protein family. Colored boxes below the black lines represent predicted Pfam domains, with the defining kinase domain shown in green, transmembrane regions are marked with black boxes and disordered regions are shown in red.

STPKs are typically signal transducers that act on response to various environmental factors. The signal is usually detected via additional domains that are tethered to the kinase domains. Binding of regulatory factors to the sensor domains leads to a conformational change in the kinase domain, which activates the signaling cascade. In the pkn family, the kinase domain, that is located in the N-terminal region of these proteins, gives similarity to eukaryotic protein kinases. The other sequence parts are specific for each of the pkn protein in MTB. With the exception of pknG and pknK, all of these proteins are highly probable to be localized to the membrane. Furthermore, members of the pkn family exhibit a significant amount of disorder and contain a large number of disordered binding regions. The location of domains, disordered segments and the transmembrane regions are shown on Figure 5.

Reflecting the functional diversity of this family, members of the pkn family are different structurally as well. Atomic level information is available for the pknB, pknD and pknG proteins. pknB contains four PASTA domains which are believed to bind peptidoglycan fragments [51], [52]. In addition, the protein is also involved in the regulation of cell shape and growth [53]. pknD encompasses 6 NHL domains forming an extracellular sensor domain. These domains were shown to fold into a highly symmetric six-bladed β-propeller [54]. In the case of pknD, ligand binding was shown to be linked to phosphate transfer. The soluble pknG protein consists of a rubredoxin and a tetratricopeptide (TPR) domain flanking the kinase domain [55]. The rubredoxin domain was found to be essential for the function and might be responsible for regulating the activity of pknG depending on the redox state of the environment. The function of the TPR domain in this case is unknown, but TPR repeats are commonly involved in variety of functions such as extensive protein-protein interaction in the assembly of multiprotein complexes in other bacterial kinases [56]. pknG was experimentally shown to be essential for avoiding the degradation of MTB cell in macrophages by disrupting the fusion of MTB with lysosomes, albeit the exact mechanism is still unknown [57].

For the other members of this family, basically very little structural information is available. Significant amount of disorder was predicted in the case of pknA, pknF and pknI. The pknA protein was reported to be involved in cell elongation, growth and division and a wide range of biological processes including positive regulation of DNA binding and negative regulation of lipid biosynthesis [58]. In contrast to the disordered pknF, pknE is likely to include an extracellular compact domain. Despite these structural differences, both kinases were reported to be involved in membrane transport [59]. pknE is also known to be linked to nitric acid stress response [60], while pknF is linked to the regulation of glucose transport and the barrier septum formation [61]. pknH is involved in transcriptional regulation and in the regulation of lipid biosynthesis [62]. Furthermore, it plays a role in the response to stress and host immune response. The functions of pknI, pknJ and pknL are largely unknown, however, pknI was hypothesized to be involved in cell division [63] and there is some indication to the involvement of pknL in transcription [64]. The largest, other soluble member of the pkn family, pknK also encompasses a large, uncharacterized structured region, C-terminally of the kinase domain. Although the structure and precise function of this region is unknown, the protein is involved in the regulation of transcription factor activity [65].

#### PE/PPE protein family

PE and PPE proteins represent the most variable group of proteins in pathogenic mycobacteria [66], [67]. The PE/PPE protein family contains 167 members and can be further divided into the PE, PE-PGRS and the PPE protein groups (with 35, 64 and 68 members, respectively) (Table S5). Despite their importance, these proteins comprise a yet greatly unexplored area as both structural and functional data concerning them are scarce.

**Figure 6:**
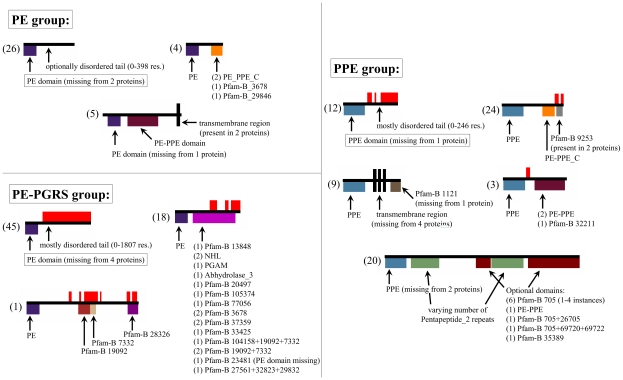
PE/PPE protein domain architectures. Domain architecture of the members of the PE/PPE protein family (PE, PE-PGRS and PPE). Colored boxes below the black lines represent predicted Pfam domains, red boxes above the black lines represent predicted disordered regions. Numbers in parentheses show the number of proteins belonging to the respective class.

The domain organization of these proteins was assessed using the Pfam domains and is shown in Figure 6. Almost all proteins contain a domain at the N-terminal region that defines the family (PE domains in the PE and PE-PGRS groups and PPE domains in the PPE group). All three groups have a small number of dominant domain configurations with which the majority of their proteins can be described. In the case of PE proteins, this configuration consists of a single PE domain optionally followed by a protein segment containing no known domains (26 out of 35 proteins). Similarly, most PE-PGRS proteins (45 out of 64) consist of a single PE domain followed by a protein segment of varying length. PPE group members are more homogeneously distributed between the different domain configurations, however the majority of them either contain a single PPE domain (followed by a segment of varying length), much like the PE or PE-PGRS proteins or a PPE domain followed by a PE-PPE_C domain (36 out of 68). A notable sub-group of the PPE group consists of 8 proteins, each containing a number of Pfam-B 705 domains, separated by repeats of the pentapeptide 2 domain and optionally a few other additional domains of unknown function (these proteins are also termed PPE-MPTR). The function of both the Pfam-B 705 domain and the pentapeptide repeats are unknown. However, as these modular proteins represent the longest members of the PE/PPE family ranging from 714 to 3300 residues in length, their structural and functional characterization is definitely of importance.

Figure 6 also shows the predicted disordered regions in the members of the PE, PE-PGRS and PPE groups. It is clear that protein disorder is not homogeneously present in all three groups. The majority of the disordered regions can be found in the PE-PGRS proteins. Although most disordered parts do not include any predicted Pfam domains, some domains significantly overlap with these regions. For example Pfam-B 33425, 20497, 37359, 13848 and 77056 domains seem to be almost entirely disordered. On the other hand, some domains, such as the α/β hydrolase domain (Abhydrolase_3), the Pfam-B 3678, 32211 and 3678 domains seem to be entirely ordered and hence might lend themselves to traditional structure determination possibly yielding potential drug targets.

In an extensive comparative genomics study, it was shown that PE and PPE genes evolved within the ESAT-6 gene cluster which codes for an entire machinery to secrete potent T-cell antigens [68]. In accordance with this, a PE protein could be identified in MTB cell culture filtrates as a proof of secretion [69]. In vivo essentiality screens showed that several of the PE/PPE proteins are essential for growth in infected mice [70]. These same proteins are coded within an ESAT-6 genomic region involved in pathogenicity [68]. Several reports also point to the fact that members of the PE and PPE families are transcribed together and function as heteromers on the cell surface [68], [71]–[73]. Several of these proteins were shown to elicit a potent T- or B-cell immune response [72], [74], [75]. Due to the variability of their C-terminal region and their sequential properties prone to mutagenesis, the PE-PGRS proteins in particular are regarded as a possible source of variable surface antigens which provide a means to exploit and possibly escape the host immune system during pathogenesis [66], [67], [71]. Therefore, it is logical to propose the inclusion of such proteins into multigenic vaccines. The cross-protection against MTB has already been shown after priming the immune system with a PE antigen containing T-cell epitopes [74]. Although the exact function of none of the PE/PPE proteins or of their complexes has been revealed, the above findings delineate a consistent picture which suggests that the PE/PPE proteins are involved in a highly plastic host-pathogen interaction network.

### Implications for target selection in drug design

In this work we carried out a comparative genomic study based on the content of domains and disordered regions and the result of large-scale sequence similarity searches. This approach is completely general and could be applied to any kind of organism with an annotated genome. Here, we focused on MTB, the causative agent of tuberculosis. Our analyses revealed two protein families in the proteome of MTB that stand out in several aspects. These proteins were also shown to have a functional importance essential for the survival of this pathogen. Next, we will examine them as potential targets for drug design.

The common properties of both the pkn and PE/PPE families include unusual domain accretions specific to this organism. This is combined with an increase in their disorder content. Both families carry out important functions in the MTB and are involved in the interactions with the host cell. Various members were shown to be essential for the organism and according to a recent analysis using guinea pig model, representatives of these families are significantly enriched in the early and chronic stages of infections [76]. Furthermore, many of them are either located in the surface of the bacteria or are exported into the host cell. The properties of these protein families underscore their biological importance and suggest that they would be ideal candidates for drug design. However, conventional drug design procedures generally overlooked such proteins as targets by largely focusing on metabolic processes. The need for novel drugs for the treatment of MTB forces researchers to explore new directions for target selection. The pkn and PE/PPE families, through their complex architectures offer several options in this regard.

One of the most active areas of therapeutic research involves developing protein kinase inhibitors. With recent advances, protein kinases have now become the second most important group of drug targets after G-protein-coupled receptors [77]. Currently, three protein-kinase inhibitors are already in clinical use, and several other protein-kinase inhibitors are undergoing human clinical trials, mostly targeting cancer [77]. However, kinases may also be exploited in the development of novel antibiotics against mycobacteria. *Mycobacterium tuberculosis* contains 11 eukaryotic-like Ser/Thr protein kinases (STPKs). All of these proteins accommodate the common catalytic domain and the 12 conserved motifs that define the signature of eukaryotic protein kinases. Despite these similarities, the sequence identity to the human SPTK genes is around 30%, low enough to allow the promising search for species-specific inhibitors [26]. Recently, a high-resolution 3D structure of the pknG kinase in complex with a potent antimycobacterial compound was published [55]. This structure revealed that despite similarities in the overall fold of the conserved kinase domain, the species-specific structural characteristics of the protein still allows highly specific interaction patterns with the promising drug-like compound. The structure of the catalytic domain of pknB in complex with different ATP analogues was also published [78], [79] and revealed that in contrast to the similar kinase structures from eukaryotes, an important regulatory loop shows high degree of flexibility in the mycobacterial kinase preventing its localization in the crystal structure. Dimerization of signaling kinases is a frequently observed phenomenon, and within the architecture of the dimer interfaces, species-specific and conserved elements are both found [80], [81]. Specifically, the dimerization of the mycobacterial STPK proteins seems to be universal and functionally relevant [82], therefore the identified intermolecular interfaces may present a further target surface to perturb protein function.

The additional sequence regions tethered to the common kinase domain are specific for each of the pkn proteins. As most of these domains are located outside of MTB cells, they could be more accessible for potential drugs. Although there is detailed structural information available for pknB, pknD and pknG proteins, they offer very few clues about potential “druggable” sites. According to their disorder profiles (Figure 5), pknE, pknK and to some extent pknJ are likely to contain additional ordered domains. The future structural characterization of these regions could offer further drug target sites. Concluding on the question whether mycobacterial kinases may present useful targets against MTB, we wish to emphasize that due the fact that many critical cellular processes in human are regulated by kinases, cross-reactivity could have dramatic consequences [83], [84]. In order to alleviate such effects, we propose to focus on a combined approach which allows targeting different elements of a mycobacterial kinase (eg dimerization domain). In such an approach, targeting the MTB-specific active site components of a kinase (eg. pknG), may be coupled to designing potentially inhibitory small molecular compounds that bind to MTB-specific sequence elements outside the active site.

The PE/PPE proteins are similar to pkn proteins in the sense that they also contain a well-conserved N-terminal domain that defines these families and C-terminal parts that show large variations in terms of their length, domain composition and disorder content. Despite the obvious biological importance of these proteins, very little structural and functional information is available for them. In a large-scale structural genomics approach it was found that individual members of both PE and PPE families did not express well or expressed in insoluble or unfolded forms [73]. To explain the failure of structure determination efforts it was suggested that these proteins need partners to fold. Indeed, via a genomic analysis one specific pair of PE and PPE proteins was predicted to interact, and their structure was successfully determined [73]. The extensive interface formed between the PE and PPE domains, that also contain the conserved N-terminal motifs, can also be considered from the viewpoint of drug design. This is supported by the growing number of examples of small molecules successfully blocking protein-protein interactions [85]. PE/PPE proteins also contain several additional ordered domains (Figure 6), however, they lack detailed structural information. In most cases, functional and structural information cannot be inferred from sequence homologs, as the family itself is highly specific to Corynebacterineae with the vast majority of PE/PPE members being present in mycobacteria only. There are, however, some exceptions, like the α/β hydrolase 3 domain, that is a catalytic domain found in a very wide range of enzymes [86], or the phosphoglycerate mutase (PGAM) domain that catalyses reactions involving the transfer of phospho groups between the three carbon atoms of phosphoglycerate [87].

Another common property of several members of both families is the presence of long disordered segments. Until recently, the feasibility of targeting proteins without a well-defined structure was unclear for the purpose of drug development. There is now, however, a newly sparked interest in intrinsically disordered proteins as potential drug targets [88], [89]. This originates partly from recognizing the biological importance of disordered proteins, especially in signaling and regulatory processes, but also from realizing the specific mode of their binding [32], [90]. Disordered proteins usually interact via a coupled folding and binding process that involves a transition from a largely flexible state to a more ordered state [34]. This transition is associated with a large entropy cost that can make the overall binding quite weak while maintaining specificity. The low binding free energy of these interactions indicates that they would be relatively easy to block by small molecules [20]. In one example, a promising small, drug-like molecule was found to bind into the groove of MDM2. This molecule inhibits the association of the ordered protein MDM2 with the disordered segment of p53 by mimicking the short alpha helical structure of p53 adopted upon binding. In another example, dimerization of two disordered proteins, c-Myc and Max, was blocked by specific inhibitors [89]. Generally, the analysis of known examples of the druggable regions of disordered proteins indicated that these segments overlapped with the binding regions predicted by ANCHOR [91]. Therefore, ANCHOR [41] and other disordered binding region prediction algorithms that will be hopefully developed in the years to come can be extremely useful to highlight potential druggable sites directly from the amino acid sequence, especially in combination with other methods. According to our results, both pkn and PE/PPE protein families contain putative druggable sites located in disordered segments. For example, the region in the C-terminal part of pknA (residues 419–431) can be of special interest, as here a high-confidence disordered binding region is predicted by ANCHOR that coincides with a high confidence predicted α helical region predicted by PSIPRED [92] (Figure S1). This is indicative of a special class of disordered binding regions that form an α helix upon binding to their partner, similarly to the binding of p53 to MDM2. The large number of disordered binding regions predicted in the PE/PPE families indicates that there can be many other druggable sites awaiting further characterization.

The two families highlighted in this study offer various options for drug design. However, most members would be omitted from traditional target selection procedures due the lack of essentiality. In the pkn families, only pknA, pknB and pknG were shown to be essential, while essentiality was showed for only 9 members of the PE/PPE family. Since MTB is an obligate parasitic pathogen, it is extremely difficult to identify genes that are required for the optimal growth of mycobacteria under in-vivo conditions. Depiction of the MTB proteome during infection is usually based on a hypothesized environment that simulates the conditions within the infected lung and defined on the basis of bacterial response to pH, starvation and hypoxia [93]–[96]. Recently, the guinea pig model was used to examine the bacterial proteome in vivo during the early and chronic stages of the disease [76]. According to this study, various members of the PE/PPE families and pknA were observed among the most dominant proteins in the infected lung samples, giving further support for the importance of these protein families. Interestingly, there were major differences between the results of these in vitro and in vivo studies, suggesting that none of the simulated in vitro model environments accurately reflects the protein profile within the lung [76]. A further limitation of studying the essentiality of individual proteins arises from the functional overlap among members of various protein families. Each member of the pkn group contains the kinase domain, and they share many of their substrates despite the differences in their sensor domains [97]. Although PE/PPE proteins are much less well-characterized, several members of them can also exhibit significant similarities with each other and this phenomenon is also reflected in their domain composition. The similarity often goes beyond the common N-terminal domain, as many members of the PE/PPE protein family share the same domain architecture (see Figure 6). There is a high likelihood of functional overlap in these cases. Protein families with overlapping functions challenge the notion of essentiality as target selection criteria. However, by targeting the common domain of protein families, several proteins of the pathogen can be attacked using the same drug molecule. Relying on such multi-target drugs can be a more efficient avenue in drug design [98]. In this respect, relatively conserved domains that occur multiple times can be of potential interest. Such examples include the PE and PPE domains, the kinase domain of pkn proteins, the NHL domain that occurs twice in the PE-PGRS groups as well as in pknD. There are several other currently uncharacterized domains occur multiple times in the genome of MTB (Figure 6). These proteins can provide interesting targets for polypharmacological drugs [18].

### Conclusions

The increasing number of complete genome sequences has enabled comparative genomic analyses which can be used to understand the distinctive properties of various pathogens and to specifically target them based on this knowledge. In this work we analyzed the proteins encoded by the genome of MTB from this viewpoint. We identified two protein families, the pkn and PE/PPE, that showed unusual species-specific enrichment of domains. These proteins can be considered as potential targets for drug design as they are involved in vital functions that are specific to this pathogen. Members of both families have complex domain architectures that combine family-specific domains with other domains and disordered segments. The analysis presented in this study predicts that drug design against members of these two families may lead to promising hits; verification of this prediction awaits further studies. It is important to emphasize that members of these two families represent novel potentials since the compounds that are either currently used in the clinics against tuberculosis or are under clinical trials are directed against other target proteins.

Although some of our findings are specific to MTB, there are several more general implications of this study. The exclusivity of certain proteins to a given pathogen is often one of prime criteria used in various target selection protocols. However, our results indicate that species-specific functions are not necessarily brought about by species-specific proteins. In contrast, many novel functions developed from already existing proteins. In the case of eukaryotes, there are several notable examples, such as the development of olfaction, reproduction, and immunity [27], where the combination of gene duplication, divergence and recombination led to the expansion of protein families and provided jumping points in evolution. The example of MTB shows that such complex evolutionary scenarios play important roles in prokaryotes as well and can be detected by species-specific enrichment of certain protein domains or families. Protein families emerging as a result of such processes often have complex domain architectures. Consequently, these proteins can be approached from multiple directions for the purpose of drug development. Besides traditional strategies that aim to inhibit enzymatic functions, less well-established approaches should also be considered, such as developing compounds against protein-protein interaction sites or disordered binding regions. Due the potential overlap within these protein families, individual members might not comply with essentiality. In these cases, however, a single drug can be effective against more than one protein. These cases of functional overlap arising from direct similarity, unlike indirect effects [99], may be predicted based on target similarity. Taking the various factors of our findings into account can help to improve the success rate of target selection protocols and drug development process.

## Methods

### Pfam

For the domain assignment the protein domains contained in the Pfam database were used (http://pfam.sanger.ac.uk/) [30]. Pfam is composed of two parts, Pfam-A and Pfam-B. Pfam-A domains are manually curated and have a distinct name (e.g. Abhydrolase_3). On the other hand, Pfam-B domains are automatically assigned using sequence similarity searches and usually no additional annotation is supplied concerning them. Pfam-B domains are not given a separate name and can be identified by a unique numerical ID.

### Sequence Dataset of Complete Proteomes (SDCP)

For the proteome-scale comparative studies, a dataset containing 1,904,578 protein sequences from 467 known complete proteomes was assembled (20 eukaryotic and 447 bacterial proteomes containing 392,401 and 1,512,177 proteins respectively). These proteomes were taken from the UniProt ftp server (ftp://ftp.uniprot.org/) [100].

### PSI-BLAST similarity searches and similarity profiles

For the similarity searches between MTB proteins and the proteins in the SDCP, PSI-BLAST was used [101]. First, a PSI-BLAST profile was calculated for each of the 3,948 proteins in the MTB proteome using the UniRef90 database, with three iterations. Next, these profiles were used to find hits from the proteins in SDCP, the database containing protein sequences from 467 known complete proteomes. A hit was considered significant (the MTB and the other protein was considered similar) and was used further on, if the e-value was below 10^−4^. Since the similarity between proteins is often restricted to shorter parts of the sequence, an explicit coverage threshold was not included in this work. This enabled the recognition of similar domains or other local protein regions between proteins, and was necessary for the successful clustering of related proteins, such as the PE/PPE families. The e-value cutoff ensured that these similarities are still significant. Based on the alignments, all locally similar sequences from the SDCP were collected for each protein in the MTB proteome. Next, for each MTB protein a similarity profile was built that contains the number of similar sequences for each of the 467 organism in the SDCP.

In general, the locally similar segments identified by PSI-BLAST are larger than single domains. The aligned regions can also contain disordered regions that are not excluded by the low complexity filter. Furthermore, since the similarity searches were centered around the MTB proteins, this analysis could also find similarities between sequences in some cases, even if they do not share Pfam domains.

### Functional assignment

For the functional categorization of MTB proteins, data were taken from the TubercuList server (http://genolist.pasteur.fr/TubercuList/) [42]. According to this site, each MTB protein is unambiguously grouped into one of the following categories: virulence, detoxification, adaptation; lipid metabolism; information pathways; cell wall and cell processes; insertion sequences and phages; PE/PPE; intermediary metabolism and respiration; regulatory proteins; conserved hypotheticals. We omitted the unknown category that contained only 16 proteins and the category corresponding to RNAs. Therefore, nine functional categories were used in this study.

### IUPred and ANCHOR

For the prediction of protein disorder IUPred was used (http://iupred.enzim.hu/) [39], [40]. The algorithm assigns a score between 0 and 1 for every residue in the protein. This score shows the tendency of that residue being disordered. For the binary categorization of residues we consider a residue disordered if it has a score greater than 0.5, and ordered if its score is less than 0.5. For the prediction of disordered binding regions we used ANCHOR (http://anchor.enzim.hu/) [37], [41]. Similarly to IUPred, ANCHOR calculates the tendency of each residue being in a disordered binding region. For binary classification, a cutoff of 0.5 was used here as well.

### Cluster analysis

The input for the clustering algorithm is based on the similarity profiles generated for each MTB sequence. Each profile consisted of 467 numbers that represent the number of sequences similar to the MTB sequence in the 467 studied proteomes. In the cluster analysis Euclidean distance was used together with Ward's method. The result of clustering was largely insensitive to various parameters of the clustering, including the type of the clustering method, various types of normalizations, parameters of PSI-BLAST. The clustering was implemented in the R program package.

## Supporting Information

Figure S1 **pknA protein predictions.** Structure predictions for the pknA protein, including IUPred, ANCHOR and PSIPRED. In the top part the kinase domain is shown in green, the transmembrane region is shown in black and disordered regions predicted by IUPred are shown in red. Solid black and striped boxes in PSIPRED predictions indicate predicted α helixes and β strands. Disordered binding regions predicted by ANCHOR are shown in blue boxes with color depth corresponding to the confidence of the prediction.
(TIF)

Table S1 **Proteins of mycolic acid synthesis.** List of proteins involved in the synthesis of mycolic acid. The corresponding functions are shown together with the name and position of the domains present. The last 5 columns show the number of occurrences of the respective domains in various groups of organisms. Bacterial specific domains are highlighted.
(XLS)

Table S2 **MTB protein profiles.** Profiles for each of the 3,948 MTB proteins obtained from the alignments performed on the CPSD (see Methods).
(XLS)

Table S3 **Proteins of each cluster.** List of the 3,948 MTB proteins. Proteins are defined with Uniprot IDs. For each protein, the number of the containing cluster is given.
(XLS)

Table S4 **pkn proteins.** List of proteins belonging to the pkn protein family. Proteins are defined with Uniprot IDs. For each protein, the pknX name is given.
(XLS)

Table S5 **PE/PPE family proteins.** List of proteins belonging to the PE/PPE protein family. Proteins are defined with Uniprot IDs. For each protein, the pknX name is given.
(XLS)
