# Supplementary figures and images for: Proteins with Complex Architecture as Potential Targets for Drug Design: A Case Study of Mycobacterium tuberculosis
Source: PLoS Comput Biol. 2011 Jul 21;7(7):e1002118. doi: 10.1371/journal.pcbi.1002118 (PMC3140968; doi:10.1371/journal.pcbi.1002118)

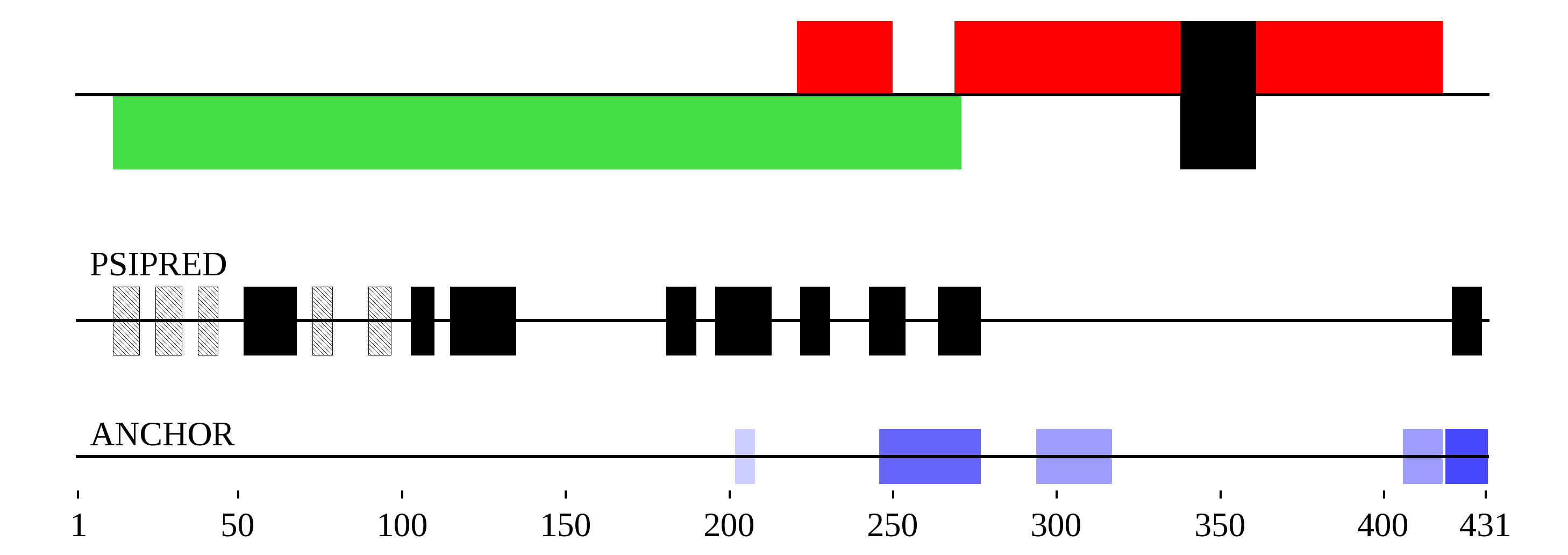

Supplement: Figure S1 — pknA protein predictions. Structure predictions for the pknA protein, including IUPred, ANCHOR and PSIPRED. In the top part the kinase domain is shown in green, the transmembrane region is shown in black and disordered regions predicted by IUPred are shown in red. Solid black and striped boxes in PSIPRED predictions indicate predicted α helixes and β strands. Disordered binding regions predicted by ANCHOR are shown in blue boxes with color depth corresponding to the confidence of the prediction. (TIF) [file pcbi.1002118.s001.tif]
